# Insights from DOCK2 in cell function and pathophysiology

**DOI:** 10.3389/fmolb.2022.997659

**Published:** 2022-09-29

**Authors:** Lulin Ji, Shuquan Xu, Haiqing Luo, Fanwei Zeng

**Affiliations:** ^1^ The Key Laboratory of Carcinogenesis and Cancer Invasion of the Chinese Ministry of Education, Cancer Research Institute and School of Basic Medical Sciences, Central South University, Changsha, China; ^2^ School of Basic Medicine, Gannan Medical University, Ganzhou, Jiangxi, China; ^3^ Organoid Research Center, Xiamen Broad Creation Biotechnology Co., Ltd., Xiamen, China; ^4^ Research and Development Center, Xiamen Mogengel Biotechnology Co., Ltd., Xiamen, China

**Keywords:** dock, cancer, ROS, migration, inflammatory response

## Abstract

Dedicator of cytokinesis 2 (DOCK2) can activate the downstream small G protein Rac and regulate cytoskeletal reorganization. DOCK2 is essential for critical physiological processes such as migration, activation, proliferation, and effects of immune cells, including lymphocytes, neutrophils, macrophages, and dendritic cells. For example, DOCK2 is involved in the development and activation of T and B lymphocytes by affecting synapse formation and inhibiting the development of the Th2 lineage by downregulating IL-4Rα surface expression. Not only that, DOCK2 may be a molecular target for controlling cardiac transplant rejection and Alzheimer’s disease (AD). Patients with defects in the DOCK2 gene also exhibit a variety of impaired cellular functions, such as chemotactic responses of lymphocytes and reactive oxygen species (ROS) production by neutrophils. To date, DOCK2 has been shown to be involved in the development of various diseases, including AD, pneumonia, myocarditis, colitis, tumors, etc. DOCK2 plays different roles in these diseases and the degree of inflammatory response has a different impact on the progression of disease. In this paper, we present a review of recent advances in the function of DOCK2 in various immune cells and its role in various diseases.

## Introduction

Immune cells are widely distributed in the body, including innate immune cells like monocytes and neutrophils and acquired immune cells such as T and B lymphocytes. Dedicator of cytokinesis 2 (DOCK2) belongs to DOCK family, which can mediate the exchange of GTP-GDP and explicitly activates the small G protein Rac1 (Miyamoto and Yamauchi, 2010). DOCK2 is predominantly expressed in immune cells, regulates actin and cytoskeleton, and mediates cell adhesion and migration (Miyamoto and Yamauchi, 2010). DOCK2 deficiency reduces the number and proportion of T cells in the lymph nodes and spleens and affects T cell development (Fukui et al., 2001; Terasawa et al., 2012). DOCK2 regulates development and activation of B lymphocytes, as well as affects plasma cell differentiation (Wang et al., 2017; Ushijima et al., 2018; Jing et al., 2019). Additionally, DOCK2 affects the cell killing function and degranulation of Natural killer (NK) cells (Sakai et al., 2013; Dobbs et al., 2015; Alizadeh et al., 2018),revealing that DOCK2 affects almost the entire process of biological action of immune cells. The development of many diseases is closely related to the activation of immune cells and the immune system, and DOCK2 is a key molecule involved in the inflammatory process involved in the development of multiple diseases. Whole genome sequencing of cancer tissues from patients with esophageal and colorectal adenocarcinoma revealed high-frequency mutations in DOCK2, suggesting that DOCK2 may play an important role in maintaining mucosal homeostasis (Frankell et al., 2019; Zaidi et al., 2020). DOCK2 has been shown to play a key role in fighting colitis caused by *Citrobacter rodentium* infection and protecting the intestine by regulating macrophage function and stabilizing the diversity of intestinal flora (Ji et al., 2021; Xie et al., 2021). DOCK2 inhibition may inhibit LPS-induced macrophage activation and may be a novel target for the treatment of endotoxemia-associated acute lung injury (Xu et al., 2021). In addition, DOCK2 may be a molecular target for controlling heart transplant rejection and Alzheimer’s disease (AD) (Jiang et al., 2005; Cimino et al., 2009; Cimino et al., 2013). Beyond immune cells, DOCK2 also affects fibroblasts. DOCK2 promotes pleural fibrosis by regulating the mesothelial to mesenchymal transition (MesoMT) leading to restrictive lung disease, as well as mediating the transition of fibroblasts to myofibroblasts (FMT) during the development of idiopathic pulmonary fibrosis (IPF) (Qian et al., 2022a; Guo et al., 2022). The alteration of TNF-α-induced, lung fibroblast (LF) pro-inflammatory phenotype in high-fat and high-fructose (HFHF)-induced pulmonary fibrosis was found to be possibly mediated by DOCK2-regulated PI3K/AKT and NF-κB pathways (Qian et al., 2022b). It is evident that DOCK2 plays different roles in different diseases, and the degree of inflammatory response has different effects on disease progression. Therefore, further understanding and exploring the function of DOCK2 in cells and clarifying the mechanism of DOCK2’s role in diseases will help provide important interventions for disease treatment. We aim to mainly present a review of DOCK2 in immune cells and various diseases.

## Structure of DOCK2

DOCK2, originally described as KIAA0209, is a member of the CDM family of proteins. It is the second DOCK protein identified in mammals and belongs to the Dock family (Fukui et al., 2001). The Dock family includes 11 family members from Dock1 to Dock11. Based on sequence and domain similarity, it is divided into four subseries DOCK-A, B, C, and D, while Dock1, DOCK2 and DOCK5 belong to the Dock-A subfamily (Miyamoto and Yamauchi, 2010). The DOCK family is very conservative in the evolutionary process. The most significant feature is that it has two domains: Dock homology region-1 (DHR1) and DHR2. All Dock proteins contain catalytic DHR2 domains with ∼450 residues located in their C-terminal region, but the DHR2 domains of the entire family are different. For example, the DHR2 domains of DOCK2 and DOCK9 have only 22% sequence homology. Among them, DOCK2 is specific for RAC and DOCK9is specific for CDC42 (Miyamoto and Yamauchi, 2010; Kulkarni et al., 2011). The DHR1 domain contains 200 ∼ 250 amino acids and can bind to phosphatidylinositol 3,4,5-triphosphate (PIP3), which is the lipid product of phosphatidylinositol 3-kinase (PI3K) (Guo and Chen, 2017; Chang et al., 2020). When PIP3 or PI3Ks were absent, Rac activation decreased (Nishikimi et al., 2009; Guo and Chen, 2017). The DHR2 domain contains 450∼550 amino acids and have guanine nucleotide exchange factors (GEFs) activity (Nishikimi et al., 2012; Chang et al., 2020). It can mediate GTP-GDP exchange, specifically activate the small G protein Rac, and regulate the formation of the cytoskeleton (Nishikimi et al., 2012). The DOCK-A and B subfamilies contain an N-terminal Src homology (SH) 3 domain and an extreme C-terminal proline sequence. On the contrary, the DOCK-D subfamily incorporates an N-terminal Pleckstrin homology (PH) domain, whereas the DOCK-C subfamily lacks a recognizable SH3 or PH domain. SH3 of DOCK2 contains 50 amino acids and can bind to Engulfment and cell motility1 (ELMO1). The SH3 domain and its adjacent α-helix region mediate its interaction with the ELMO1 subunit (Chang et al., 2020). Binding of SH3 and Elmo1 inhibits DOCK2 ubiquitination to prevent DOCK2 degradation and promotes Rac activation by binding DHR2 to Rac (Hanawa-Suetsugu et al., 2012; Nishikimi et al., 2013). Furthermore, DOCK2 promotes cell chemotaxis by binding its C-terminal polybasic amino acid region (PBR) to the cell membrane component phosphatidic acid (PA) (Gomez-Cambronero, 2010).

## Role of DOCK2 in adaptive immune cells

### DOCK2 regulates the transport and homing of lymphocytes

The transport of lymphocytes is involved in maintaining the homeostasis of the immune function of the body and is essential for the induction of an adaptive immune response. Naive lymphocytes perform normal immune functions through recirculation processes in secondary lymphoid tissues (e.g., lymph nodes, Peyer’s lymph nodes, and spleen) (Tanaka et al., 2006). T cells and B cells are regulated by chemokines (C-X-C motif chemokine 13, CXCL13), (C-C motif ligand 19, CCL19), and CCL21 to enter secondary lymphoid organs through blood circulation. After encountering antigens in secondary lymphoid tissues, most lymphocytes can migrate to extra lymphoid tissues to perform their functions (Stein and Nombela-Arrieta, 2005). Therefore, lymphocyte transport is essential for inducing adaptive immune responses in the organism. The precise specificity of lymphocyte transport is dependent on specific signals from certain chemokines. Lymphocyte migration from bone marrow or thymus to peripheral lymphoid organs is driven by chemokines such as CCL21, CXCL12, and CXCL13. On the contrary, the migration of lymphocytes from peripheral lymphoid organs to the site of infection is mediated by Sphingosine-1-phosphate (S1P) (Moser and Loetscher, 2001; Matloubian et al., 2004). Wild-type (WT) lymphocytes have been shown to activate Rac to migrate efficiently in a dose-dependent manner when stimulated with CCL21 and CXCL13 *in vitro*. However, *DOCK2* ^*−/−*^ lymphocytes did not show migration-related responses to these chemokines, including CXCL12 (T and B cells), CXCL13(B cells) and CCL21(T cell), leading to impaired homing of T and B cells in secondary lymphoid organs, as well as severe atrophy of secondary lymphoid organs (Moser and Loetscher, 2001; Terasawa et al., 2012). T cells in WT mice regulate chemokine signaling through the CCL21-DOCK2-Rac-F-actin pathway, and DOCK2 deficiency reduced Rac activation and decreased F-actin polymerization, affecting cytoskeletal rearrangements and shape changes (Nombela-Arrieta et al., 2004; Nombela-Arrieta et al., 2007). The number and percentage of lymphocytes in the spleen and lymph nodes of *DOCK2* ^*−/−*^mice were also significantly reduced compared to WT mice (Fukui et al., 2001). Furthermore, CCL21-DOCK2-Rac is the main pathway of migration of Naïve T (NT) cells into lymphoid tissues. Chemokine receptor 7(CCR7)-mediated NT motility was primarily influenced by the Rac activator DOCK2 and secondarily by PI3Kγ-dependent ways (Nombela-Arrieta et al., 2004; Nombela-Arrieta et al., 2007), treatment of NT cells with the 4-[3'-(2″-chlorophenyl)-2′-propen-1′-ylidene]-1-phenyl-3,5-pyrazolidinedione (CPYPP) inhibitor significantly reduced cell-dependent PI3Kγ pathway migration. The combination of CPYPP and AS-605240 completely blocked the migration of NT to CCL21 (Thelen et al., 2021). Among them, CPYPP inhibitor inhibits DOCK2-Rac1 interactions and AS-605240 is PI3Kγ-specific (Thelen et al., 2021). DOCK2 is also believed to be involved in chemokines-mediated integrin activation. Although *DOCK2* ^*−/−*^T cells were not affected in integrin-dependent adhesion, integrin activation of B cells was impaired, resulting in a significant reduction in integrin-induced B cell migration (Nombela-Arrieta et al., 2004). Thus, T cells and B cells may have different pathways in chemokine-induced integrin activation (Nombela-Arrieta et al., 2004). In summary, the DOCK2 protein plays a crucial role in lymphocyte trafficking and homing by regulating the cytoskeleton, cell adhesion, and migration of actin through activation of Rac. However, there is a lack of systematic understanding of the specific mechanisms involved in the regulation of DOCK2 by various factors.

### DOCK2 promotes T-lymphocyte activation, proliferation and differentiation

The actin backbone of lymphocytes is an essential mediator of the formation and maturation of immune synapses and their signaling and cellular activity (Wurzer et al., 2019). The immune synapse is the interface formed by the interaction of immune response molecules between T cells and antigen-presenting cells (APC). Once exchange is disrupted, the host may experience escape from the tumor/pathogen or be attacked by an autoimmune response (Dustin, 2014). The number of double-positive (DP) thymocytes was markedly reduced in *DOCK2* ^*−/−*^ 2B4 TCR transgenic (Tg) mice, suggesting that DOCK2 regulates the threshold for positive selection in the thymus (Sanui et al., 2003). The study finds that the mouse spleen or thymus regulates T cells *via* the T cell receptors (TCR)-DOCK2-Rac pathway (Sanui et al., 2003). Compared with *DOCK2* ^*+/−*^ T cells, the formation of the interface between *DOCK2* ^*−/−*^T cells and APCs and antigen-induced TCR translocation and lipid rafts located at the interface are severely impaired. These lead to a significant decrease in antigen-specific T cell proliferation (Sanui et al., 2003). Thus, DOCK2 promotes T cell activation and differentiation by regulating Rac activation downstream of the TCR signaling pathway and remodeling the actin skeleton (Table 1). A recent study found a decrease in basal and maximal mitochondrial oxygen consumption of T cells in two *DOCK2* ^*−/−*^patients, suggesting impaired mitochondrial function (Alosaimi et al., 2019). When TCR is stimulated, the proliferation response of cells is also disrupted (Alosaimi et al., 2019). This suggests that DOCK2 promotes T cell activation by activating Rac to maintain mitochondrial function integrity (Alosaimi et al., 2019). Furthermore, DOCK2 can maintain IL-4Rα homeostasis by activating Rac and thus regulating T cell differentiation (Tanaka et al., 2007). DOCK2-deficient CD4^+^T cells are affected by antigen-driven downregulation of IL-4Rα expression, leading to sustained IL-4 signaling and induction of Th2 type differentiation (Tanaka et al., 2007). Thus, the DOCK2 protein regulates the formation, proliferation, activation and differentiation of T lymphocyte immune synapses through Rac activation, allowing lymphocytes to function generally in adaptive immunity. CD8^+^T cells play a key role in the adaptive immune response. One of the key features of the T cell response is the establishment of long-lived memory cells that respond rapidly to re-exposure to pathogens and play an important role in preventing infection and reinfection with intracellular pathogens such as viruses (Alosaimi et al., 2019). Virtual memory T cells are not exposed to foreign antigens. They have an acquired memory-like phenotype that can advance into the cell cycle or modulate its effector functions by lowering the threshold for signaling (Hussain and Quinn, 2019). Virtual memory cells are involved in resistance to intracellular bacterial infection. Increased virtual memory CD8^+^T cell expression of DOCK2-deficient mice was associated with increased resistance to *Listeria monocytogenes*, with the higher secretion of types of interferon (IFN)-γ against intracellular bacterial after infection (Mahajan et al., 2020). Furthermore, there is a direct link between lymphocyte conversion to memory and the strength of the tensional self-peptide signal received by T cells. While DOCK2 may promote TCR responses to potent agonists, DOCK2-deficient CD8^+^T cells have enhanced responses to weak agonists (Mahajan et al., 2020). DOCK2 defects reduce the affinity threshold of cells entering the virtual memory compartment for their antigens, leading to increased conversion of naive T lymphocytes directly to virtual memory T cells (Mahajan et al., 2020). Although most DOCK2 defects of T cells have a negative impact, DOCK2 can inhibit the generation of CD8^+^virtual memory T cells by controlling the response threshold to weak agonists, which also reveals a negative regulatory effect of DOCK2 on CD8^+^T virtual memory cells. Taken together, DOCK2 not only regulates T cell migration, development, proliferation, activation, and differentiation, but also regulates the normal function of CD8^+^T memory cells.

**TABLE 1:** The role of DOCK2 in immune cells.

Immune cells	Main functions	Rho	References
T cells			
Effector T cells	Activates Rac in the TCR signaling pathway and mitochondrial function, promoting T cell proliferation and activation	Rac1; Rac2	Sanui et al. (2003), Alosaimi et al. (2019)
Helper T cells	Regulates Th2 cell differentiation by activating Rac and thus maintaining IL-4Rα homeostasis	Rac1; Rac2	Tanaka et al. (2007)
Memory T cells	Increases the cellular agonist threshold, and thereby inhibits the formation of CD8^+^ virtual memory T cells	Unknown	Mahajan et al. (2020)
B cells			
Effector B cells	Promotes the growth of BCR microclusters by remodeling F-actin; Promotes the proliferation and activation of B cells by regulating CD19 and CD21; Maintains normal IgG antibody production	Rac1; Rac2	Wang et al. (2017), Ushijima et al. (2018), Jing et al. (2019)
Memory B cells	Regulates the proliferation of memory B cells by affecting LEF-1 and HIF-1α	Rac1; Rac2	Yang et al. (2020)
Natural killer cells	Regulates the cytotoxic function of NK cells and the secretion of IFN-γ through the Rac pathway mediated by NKG2D	Rac1	Sakai et al. (2013), Dobbs et al. (2015), Alizadeh et al. (2018)
Natural killer T cells	Affects the transformational development and proliferation of Vα14 NKT cells	Unknown	Kunisaki et al. (2006a)
Neutrophils	Promotes neutrophil polarization and chemotaxis by activating Rac and affecting F-actin production	Rac1; Rac2	Kunisaki et al. (2006b), Liu et al. (2016), Moens et al. (2019)
Macrophages			
M1	Promotes pro-inflammatory cytokines from macrophages and increases MPO activity	Rac1	Xu et al. (2021)
Unknown	Induces macrophage migration by regulating chemokines; increases ROS production and affects phagocytosis and bactericidal functions	Rac1	Guo et al. (2017), Ji et al. (2021), Ma et al. (2022a)
Dendritic cells pDCs	Regulates pDC type I interferon production and migration through the TLR7/TLR9 pathway	Rac1	Gotoh et al. (2008), Gotoh et al. (2010), Chu et al. (2019)

### DOCK2 promotes B-lymphocyte activation, proliferation and differentiation

As PI3K and Phosphatase and tensin homolog deleted on chromosome 10 (PTEN) together maintain PIP3 production, PIP3 binds to DOCK2 to activate Rac to promote F-actin remodeling for sustained growth of BCR microclusters and the formation of immune synapse (Wang et al., 2017). Consistent with this, defects in DOCK2 lead to structural disruption of B cell immune synapses. *In vitro* stimulation of B cells or B cell transmigration experiments demonstrated that B cells mediate BCR signaling through the DOCK2-Rac axis, promoting the formation of immune synapse and plasma cell differentiation (Ushijima et al., 2018). In B cell specific DOCK2 knockout mice, serum Immunoglobulin (Ig)G1 and IgG2b levels were significantly lower and specific IgG antibody production was severely impaired compared to control mice (Ushijima et al., 2018). Thus, DOCK2 regulates the development and activation of B lymphocytes through Rac activation. Interestingly, another study demonstrated that DOCK2-pWASP-F-actin promotes marginal zone (MZ) B cells from WT mice for sustained growth of BCR microclusters (Jing et al., 2019). Recent studies have also found that the number of MZ B cells in *DOCK2* ^*−/−*^mice is significantly decreased compared with C57BL/6 J mice due to a decrease in Phosphorylated Wiskott–Aldrich syndrome protein (pWASP) caused by the absence of DOCK2, which allows the upregulation of the transcription factor lymphoid enhancer-binding factor (LEF)-1, thus suppressing CD21 expression at the mRNA level (Jing et al., 2019). The absence of DOCK2 also led to a decrease in CD19 expression (Jing et al., 2019). Additionally, DOCK2 also regulates memory B cells through the LEF-1-CD21-CD19 pathway, resulting in reduced expression of CD19 and CD21 in memory B cells in DOCK2-deficient patients, reducing the number of memory B cells and disrupting early B cell activation (Yang et al., 2020). Therefore, CD21 and CD19 mediate downregulation of BCR signaling and reduction of early B-cell activation (Jing et al., 2019; Yang et al., 2020). DOCK2-deficient B cells also exhibit defects in endoplasmic reticulum (ER) and mitochondrial function. DOCK2-deficient patient B cells, *DOCK2* ^*−/−*^mice B cells and A20 cells knocked down for DOCK2 demonstrated that DOCK2 affects the ER of B cells and mitochondrial structure, which induces HIF-1α activity, enhances B cell metabolism, and ultimately leads to increased apoptosis (Yang et al., 2020). Therefore, the findings suggest that the reduced number of memory B cells in DOCK2-deficient patients may correlate with increased cell metabolism. Taken together, DOCK2 is essential for the formation, development, and activation of immune synapses of B lymphocytes.

## Role of DOCK2 in innate immune cells

### DOCK2 regulates NK cell cytotoxicity, degranulation, IFN-γ production and NKT cell development

NK cells are innate immune cells that function in fighting tumors and viral infections and promote or inhibit the function of other immune cells by secreting cytokines or chemokines. Excessive activation or dysfunction of NK cells may be related to the pathogenesis of certain diseases (Zhang and Tian, 2017). The Natural killer group 2 member D (NKG2D) receptor is one of the receptors of NK cells and recognizes the endogenous major histocompatibility complex (MHC)-I (Raulet, 2003). The engagement of NKG2D is a sufficient stimulus to activate cytolysis and cytokine production by NK cells (Raulet, 2003). Studies showed that cytotoxicity and IFN-γ secretion were significantly lower in *DOCK2* ^*−/−*^mice NK cells compared to WT mice (Sakai et al., 2013). Although *DOCK2* ^*−/−*^NK cells can bind to target cells *in vitro*, they do not effectively kill leukemic cells or act on MHC-I-deficient myeloid cells. PI3K activity is required for NK cell-mediated cytotoxicity and synapse formation (Sakai et al., 2013). Studies show that stimulation of NKG2D induces the accumulation of PIP3 and DOCK2 binds to PIP3 through the DHR-1 domain (Sakai et al., 2013). Thus, DOCK2 may be transferred to the synapse through the interaction of the DHR-1 domain with PIP3. In conclusion, DOCK2 regulates the cytotoxic function of NK cells and the secretion of IFN-γ through the NKG2D-mediated Rac activation pathway (Sakai et al., 2013). CD4^+^T cells, CD19^+^B, and CD16/CD56^+^NK cells are reduced in a rare DOCK2-deficient patient with significantly elevated IgM (Alizadeh et al., 2018). Furthermore, a clinical study involving five children with a double allele mutation in DOCK2 showed reduced lymphocytes and impaired T, B, and NK cell responses in these children compared to healthy controls, with defects in NK cell degranulation, actin polarization, and extracellular signal-regulated kinase (ERK) signaling pathways (Dobbs et al., 2015). Briefly, DOCK2 regulates NK cell cytotoxicity, degranulation and IFN-γ secretion. NKT cells act as a bridge between innate and adaptive immunity and have the expression of TCR and NK cell lineage receptors (Cui and Wan, 2019). The number of NKT cells in the thymus, spleen and liver of *DOCK2* ^*−/−*^mice was significantly reduced compared to WT mice (Kunisaki et al., 2006a). Mouse CD1d-restricted Vα14 NKT cells are a distinct lymphocyte subpopulation vital for tumor surveillance and host defense against pathogens (Kunisaki et al., 2006a). *DOCK2* ^*−/−*^mice NKT cells stimulated with Vα14NKT ligand showed little detectable cytokine production, and DOCK2-deficient Vα14NKT cells were impaired in early development relative to control cells (Kunisaki et al., 2006a). The number of peripheral blood NKT cells was also significantly reduced in children with DOCK2 double allele mutation (Dobbs et al., 2015). These results suggest that DOCK2 may affect the development of transformation and proliferation of T-cell precursors to Vα14 NKT cells, but the exact mechanism is currently unknown.

### DOCK2 regulates neutrophil migration, ROS production and NETs formation

Neutrophils are the main population of leukocytes involved in the inflammatory response in response to tissue injury or the occurrence of infection. They are recruited to the site of inflammation to participate in the innate immune response through the induction of chemical elicitors. Chemical elicitors include lipids, N-formylated peptides, complements, allergenic toxins, and chemokines. Recognition and phagocytosis of microorganisms through chemotaxis and release of mediators by neutrophils after binding of chemical elicitors to the G protein-coupled receptor (GPCR) (Petri and Sanz, 2018). Compared to WT, fMLP and PMA-induced Rac1/Rac2 activation in *DOCK2* ^*−/−*^neutrophils was significantly reduced, as was reactive oxygen species (ROS) production (Kunisaki et al., 2006b). And the formation of neutrophil extracellular traps (NETs), which are dependent on ROS production, was impaired after activation by chemotactic agents (Watanabe et al., 2014). NETs are DNA backbone, intercalated proteins with bactericidal and increased permeability functions. Activated neutrophils can form NETs to capture and destroy pathogens and participate in the antimicrobial action of the body (Brinkmann et al., 2004). N-formylmethionyl-leucyl-phenylalanine (fMLP) can be produced by bacteria (e.g., *Escherichia coli* and *Staphylococcus aureus*) (Marasco et al., 1984)and strongly stimulate the chemotactic response of neutrophils (Atkinson et al., 1988; Campbell et al., 1997). Microscopic analysis observed induction of WT and *DOCK2* ^*−/−*^ neutrophils by fMLP at 30 s. WT neutrophils exhibited localized accumulation of F-actin at 30 s, while *DOCK2* ^*−/−*^neutrophils showed almost none, although partially recovered after 60 s (Kunisaki et al., 2006b). Consistent with this result, most *DOCK2* ^*−/−*^neutrophils in the chemotactic state exhibited abnormal morphology and a narrower distribution of F-actin, suggesting that F-actin accumulation requires DOCK2 activation. *In vivo* experiments also demonstrated defective migration of neutrophils from the submucosa to the lamina propria of the colon in *DOCK2* ^*−/−*^mice during *Citrobacter* infection (Liu et al., 2016). Defective neutrophil cytoskeletal rearrangements and shape changes and reduced F-actin polymerization, which are required for neutrophil polarization and chemotaxis, were also found in patients with mutations at the DOCK2 shear site (c. 2704-2A > A) (Moens et al., 2019). Thus, DOCK2 plays a very important role in neutrophil migration, ROS production, and NET formation.

### DOCK2 regulates macrophage migration, activation and ROS production

Macrophages can be divided into classically activated macrophages (M1) and alternatively activated macrophages (M2), with sophisticated continuous subtypes due to stimuli and microenvironments (Hu et al., 2021). M1 macrophages produce inflammatory factors involved in pro-inflammatory immune responses and play an important role in repairing tissue injury sites and the development of inflammatory-related diseases (Hu et al., 2021). LPS stimulation has been shown to promote the release of pro-inflammatory cytokines through the Rac-NF-kB pathway by regulating the activation of M1-type macrophages through DOCK2 (Xu et al., 2021). Since LPS stimulation enhances the expression of M1-type macrophage DOCK2 *via* the Rac-NF-kB pathway, it controls the activation of IκB kinaseβ (IKKβ). It promotes the release of pro-inflammatory cytokines IL-6, TNF-α, and IL-1β. Both shDOCK2 and EHop-016 resulted in impaired Rac activation, reduced pro-inflammatory cytokine release, and decreased myeloperoxidase (MPO) activity in response to LPS stimulation (Xu et al., 2021). To add, EHop-016 is a Rac inhibitor. Injection of the CPYPP inhibitor significantly inhibited macrophage infiltration, attenuated the release of pro-inflammatory cytokines IL-6, TNF-α, and IL-1β, and considerably reduced MPO activity in an endotoxin-induced mouse model (Xu et al., 2021). Therefore, DOCK2 can promote the release of pro-inflammatory cytokines and increase MPO activity by activating Rac-mediated increase in NF-kB. Unlike WT mice, *DOCK2* ^*−/−*^mice were found to have reduced macrophage infiltration and reduced inflammation after eating chow or high-fat diet (HFD), including IL-6, TNF-a, MCP-1, and IL-12, all of which were affected and expressed in reduced amounts (Guo et al., 2017). Other studies have also shown defective migration of macrophages from the submucosa to the lamina propria of the colon in *DOCK2* ^*−/−*^mice during *Citrobacter* infection (Liu et al., 2016). Further exploration revealed that in bone marrow-derived macrophages (BMDMs) from *DOCK2* ^*−/−*^ mice, when stimulated with CXCL12,CCL4, and CCL5, the migration capacity was compromised compare to that from WT mice (Ji et al., 2021). As demonstrated by *in vitro* bacterial infection experiments, *DOCK2* ^*−/−*^BMDMs showed decreased expression of chemokines and chemokine receptors and impaired phagocytosis and bactericidal capacity after bacterial stimulation. However, BMDMs from *DOCK2* ^*−/−*^ mice had defects secretion of chemokines CCL4 and CCL5 after infection, which was partially restored upon adoptive transfer of wild-type BMDMs (Ji et al., 2021). Thus, DOCK2 plays an important role in mediating the migration, phagocytosis, and bactericidal functions of macrophages. Macrophages also play an essential role in antifungal immunity. Studies have shown that DOCK2 has an indispensable role in natural antifungal immune signaling and pro-inflammatory gene expression (Ma et al., 2022a). Using *Candida albicans* for systemic fungal infection in WT and *DOCK2* ^*−/−*^mice, a defective antifungal response to systemic fungal disease was found in *DOCK2* ^*−/−*^mice compared to WT, with significantly reduced infiltration of macrophages and neutrophils in the kidney (Ma et al., 2022a). C-type lectin receptors (CLRs) can be recognized by innate immune cells through different binding components of the fungal cell wall such as β-glucan, α-mannose, mycelial mannose, and glycolipids, thus initiating antifungal immune responses (Ma et al., 2022a). *DOCK2* ^*−/−*^BMDMs induced with *Candida albicans*, α-mannose or Curdlan showed significantly lower pro-inflammatory cytokine and chemokine expression than WT BMDMs (Ma et al., 2022a). Curdlan, a β-1,3-glucan isolated from *Alcaligenes faecalis*, is an agonist of dectin-1 in immune cells (Kim et al., 2016). And DOCK2-deficient macrophages exhibited reduced Rac1 activation and ROS production after fungal stimulation, resulting in decreased macrophage inflammatory gene expression and bactericidal activity (Ma et al., 2022a). Mechanically, the interaction between the SH3 structural domain of DOCK2 and the C-terminus of SYK allows the phosphorylation of DOCK2 at tyrosine 985 and 1405, facilitating the recruitment and activation of Rac GTPases in the cell membrane. This increases the production of reactive oxygen species to activate macrophage signaling and fungicidal activity (Ma et al., 2022a). In summary, DOCK2 plays a crucial role in macrophage migration, phagocytosis, bactericidal, and ROS production.

### DOCK2 regulates the antiviral function of pDCs cells

Dendritic cells are classified into myeloid dendritic cells (mDCs) and plasmacytoid dendritic cells (pDCs) based on their morphology, cell surface markers, and functions (Shortman and Liu, 2002). mDCs primarily act as antigen-presenting cells, delivering antigens to T cells, while pDCs primarily produce IFNs, which are essential for host defense against viral infection (Liu, 2001; Swiecki and Colonna, 2010). Although DOCK2 does not directly affect the development of pDCs, the absence of DOCK2 attenuates chemical stimulus-induced Rac activation and the migratory response of pDCs (Gotoh et al., 2008). On the contrary, *DOCK2* ^*−/−*^mDCs are not defective in Rac activation and migration, which may be due to functional compensation by Dock1 and DOCK5in cells (Gotoh et al., 2008). Because DOCK1and DOCK5are known as Rac-specific GEFs. Type I IFNs were shown to be significantly reduced in *DOCK2* ^*−/−*^pDCs compared to WT mice pDCs (Gotoh et al., 2010). The induction of Rac activation after pDCs exposure to TLR ligand is dependent on DOCK2, but DOCK2 deficiency affects IKK-α phosphorylation and impaired nuclear translocation of Interferon Regulatory Factor 7(IRF-7), leading to a decrease in IFN-α (Gotoh et al., 2010). When RNA and DNA recognize TLR7 and TLR9, respectively, pDCs produce not only inflammatory cytokines but also a large amount of IFN (Chu et al., 2019). Thus, activating the Rac pathway by DOCK2 synergistically induces IFN-α production by pDCs *via* the TLR recognition microbial structure pathway (Chu et al., 2019). Although the exact mechanism of how nucleic acid ligands activate Rac is not yet clear, DOCK2 may be involved in producing type I IFNs in pDCs, thus enhancing their antiviral function.

## Role of DOCK2 in diseases

### DOCK2 regulates microglia function to promote the development of Alzheimer’s disease and cerebral ischemia/reperfusion

Innate immune activation of the central nervous system is associated with several neurodegenerative diseases, including AD, and the main cellular component is activated microglia (Cimino et al., 2009). DOCK2 was expressed exclusively in brain microglia and almost exclusively dependent on the regulation of the prostaglandin E2(PGE2) receptor EP2 (Cimino et al., 2009). After exposing the primary microglia to 24 h of LPS, *DOCK2* ^*−/−*^microglia secreted significantly less TNF-α and MCP-1 cytokines and had substantially less phagocytic capacity compared to WT. Compared to co-cultures of neurons (WT) and microglia (WT or *DOCK2* ^*−/−*^), co-cultures with *DOCK2* ^*−/−*^ microglia after WT neuronal damage exhibited reduced (Cimino et al., 2009). Thus, DOCK2 regulates the secretion of cytokine, phagocytosis, and paracrine functions of microglia (Tables 2, 3). In the human brain, DOCK2 was also shown to be expressed almost exclusively in microglia in the human frontal cortex and hippocampus (Cimino et al., 2013). The number of *DOCK2* ^*+/+*^cells was increased significantly in the brains of AD patients compared to normal controls, and *DOCK2* ^*+/+*^ microglia were associated with Tau protein neurogenic fiber tangles and Aβ plaques. To investigate the role of DOCK2 in AD, the authors performed ablation of the DOCK2 gene in APPswe-PS1Δe9 mice (AD model) and found that the reduction in Aβ plaques occurred after ablation of the EP2 gene was largely reproduced (Cimino et al., 2013). However, soluble levels of aβ42 did not differ in *DOCK2* ^*−/−*^mice versus WT mice. Although the results suggest that the expression of microglia-specific DOCK2 in the brain is involved in the accumulation of Aβ plaques, it is not involved in the production and clearance of soluble aβ42 levels (Cimino et al., 2013). Since *DOCK2* ^*−/−*^microglia phagocytosis is significantly reduced, it is hypothesized that the increase in DOCK2-promoted plaques is likely due to a change from soluble to insoluble regulated by the inflammatory environment (Cimino et al., 2013). In summary, DOCK2 is involved in the formation of Aβ plaques by regulating the immune function of microglia. Unlike the direct influence of targeting EP2 on Cyclooxygenase (COX), its further in-depth study may be more beneficial for AD treatment. DOCK2 has also been shown to have an important role in cerebral ischemia/reperfusion (Ding et al., 2022). High expression of DOCK2 was demonstrated by establishing mouse and cell line models resulting in effects on brain infarcts and neuron degeneration. Further, DOCK2 was found to regulate the polarization of microglia. It was demonstrated that DOCK2 promotes the involvement of M1 microglia in the inflammatory response by affecting p-STAT6, which promotes the development of cerebral ischemia/reperfusion (Ding et al., 2022). In any case, starting with DOCK2 to study the mechanism of its involvement in the pathological changes of brain related diseases and the development of new drugs can provide a new idea for preventing and treating brain related diseases.

**TABLE 2:** The role of DOCK2 in diseases.

Diseases	Main subjects	Mechanisms	Functions	Immune cells involved	References
Nervous system disease					
Alzheimer’s disease	*In vivo; In vitro*	Regulates cytokine secretion, phagocytosis and paracrine neurotoxicity in microglia; Promotes the accumulation of Aβ plaques	Promotes the development of AD.	Microglial	Cimino et al. (2009), Cimino et al. (2013)
Stroke	*In vivo*	Downregulates the expression of p-STAT6, thereby promoting M1 polarization	Aggravates the cerebral ischemia/reperfusion	Microglial	Ding et al. (2022)
Respiratory disease					
Pulmonary fibrosis	*In vivo*; *In vitro*	Regulates the TGF-β-mediated MesoMT and FMT processes	Promotes the extent of pulmonary fibrosis	Macrophages	Qian et al. (2022a), Ma et al. (2022b), Qian et al. (2022b), Guo et al. (2022)
Acute lung injury	*In vivo*	Exacerbates inflammatory cell infiltration and increases MPO activity	Promotes the advancement of ALI	Macrophages	Xu et al. (2021)
COVID-19	Human samples; *In vivo; In vitro*	Regulates macrophages recruitment and IFNs response	Inhibits the progression of COVID-19		Namkoong et al. (2022)
Lung cancer	Human samples	May excessively activate the MYC and the DNA repair signaling pathways	Promotes the progression of NSCLC		Zeng et al. (2021)
Heart Diseases					
Myocarditis	*In vitro*	Reduces t miR-16 expression and upregulates the expression of pro-inflammatory factors	Facilitates the development of myocarditis		Wang et al. (2020)
Digestive system diseases					
Colitis	*In vivo; In vitro*	Regulates macrophage’s function and microbial populations bidirectionally	Inhibits the development of colitis	Macrophages	Liu et al. (2016), Ji et al. (2021), Xie et al. (2021)
Colorectal cancer	Human sample	Mediates the recruitment of T cells	Inhibits the malignant progression of CRC	Lymphocytes	Yu et al. (2015), Miao et al. (2018), Kadkhoda et al. (2021)
Prostate cancer	Human sample; *In vitro*	Involved in methylation and cell proliferation	Predicts the malignant progression of PCa		El Haibi et al. (2010), El-Haibi et al. (2012), Bjerre et al. (2019), Bjerre et al. (2020)
Hematologic Diseases					
Chronic lymphocytic Leukemia	Human sample; *In vivo*; *In vitro*	Regulates the cell proliferation together with Wnt5a-ROR1 axis	Promotes the progression of CLL	Lymphocytes	Wang et al. (2010), O'Hayre et al. (2010), Hasan et al. (2018)
Acute myelocytic leukemia	Human sample; *In vivo*; *In vitro*	Regulates the cell proliferation through Rac1-related pathway and sensitivity to drugs	Promotes the development of AML	Lymphocytes	Nishihara et al. (2002), Wu et al. (2017), Hu et al. (2019), Wu et al. (2019)
Skin cancer					
Melanoma	*In vivo*; *In vitro*	Assists melanoma stem cells to anti-apoptosis	Contributes to the development of melanoma		Chu et al. (2021), Zhang et al. (2022)
Transplantation Immunology					
Rejection of heart transplantation	*In vivo*	Regulates T cell numbers and inflammatory factor levels	Exacerbates graft rejection	Lymphocytes	Jiang et al. (2005)
Immune deficiency disease					
Combined immunodeficiencies	Human sample; *In vivo*; *In vitro*	Promotes the growth of T and B cells and ensures the function of NK cells	Maintains normal immune function	Lymphocytes	Alizadeh et al. (2018), Dobbs et al. (2015), Alosaimi et al. (2019), Sharifinejad et al. (2021), Aytekin et al. (2021), D'Astous-Gauthier et al. (2021)

*In vivo* represents animal experiments; *In vitro* represents cell experiments; Human samples represent human sample sections and human related databases.

**TABLE 3:** The role of immune cells in diseases.

Immune cells	Involved disease	Functions of cells in disease	References
Microglial	Alzheimer’s disease	Regulates the accumulation of Aβ plaques	Cimino et al. (2009), Cimino et al. (2013)
	Stroke	Affects the progression of cerebral ischemia/reperfusion	Gotoh et al. (2008)
Macrophages	Lung Injury	Promotes obesity and affects lung inflammation	Qian et al. (2022b)
	Acute lung injury	Exacerbates the level of inflammatory response	Xu et al. (2021)
	Colitis	Participate in the early inflammatory reaction	Ji et al. (2021)
	Colorectal cancer	Suppresses tumor progression	Myant et al. (2013)
Lymphocytes	Chronic lymphocytic Leukemia	Involves in the development of CLL.	Yu et al. (2016), Sakamoto et al. (2017), Hallek et al. (2018)
	Acute myelocytic leukemia	Involves in the development of AML.	Levis and Small (2003), Wu et al. (2017), Wu et al. (2019), Stoner et al. (2020)
	Rejection of heart	Plays a key role in rejection of heart	Jiang et al. (2005)
	Combined immunodeficiencies	Affects the integrity of immune function	Dobbs et al. (2015), Alizadeh et al. (2018), Wurzer et al. (2019), Kunimura et al. (2020), Sharifinejad et al. (2021)

### DOCK2 mediates inflammatory responses in chronic and acute pneumonia

DOCK2 can promote pleural fibrosis by modulating mesothelial to mesenchymal transition (MesoMT), which results in restrictive lung disease (Qian et al., 2022a). Transforming growth factor-β(TGF-β) is the most potent profibrotic factor and it can induce MesoMT *in vitro* and pleural fibrosis *in vivo* (Gauldie et al., 2007; Owens et al., 2015; Boren et al., 2017). TGF-β enhanced the expression of DOCK2 in primary HPMCs, and DOCK2 knockdown also alleviated TGF-β-induced MesoMT (Qian et al., 2022a). Compared to C57BL/6 mice, DOCK2-knockout mice are protected against *Streptococcus Pneumoniae*-induced impairment of pleural and pulmonary compliance (Qian et al., 2022a). The researchers also found that DOCK2 mediates fibroblast to myofibroblast transition (FMT) in the development of idiopathic pulmonary fibrosis (IPF) (Guo et al., 2022). IPF is the most common form of chronic interstitial pneumonia. Although its etiology is unknown, imbalance of apoptosis of alveolar epithelial cells or fibroblasts is one of the important pathogenic mechanisms (Ma et al., 2022b). TGF-β enhanced the expression of DOCK2 and the FMT marker α-SMA in primary human lung fibroblast (HLF), and DOCK2 knockdown also dramatically attenuated the TGF-β-induced expression of the FMT marker. This investigation proved that DOCK2 is induced by TGF-β *via* the Smad3 and ERK pathways in primary HLFs. The involvement of DOCK2 in the transition of lung fibroblast (LF) phenotype affecting lung disease was also confirmed in a bleomycin-induced lung fibrosis model in mice and in IPF patients. In a high-fat and high-fructose (HFHF) diet, often accompanied by the development of pulmonary inflammation and pulmonary fibrosis (Qian et al., 2022b). LFs play a key role in lung inflammation. Since *DOCK2* ^*−/−*^mice have reduced body weight and reduced inflammatory response in high-fat diet and DOCK2 deficiency significantly attenuates pulmonary inflammatory and profibrotic injury in the HFHF model (Qian et al., 2022b). After transduction of HLF by Ad-shDOCK2, decreased DOCK2 was found to significantly block the pro-inflammatory phenomenon, i.e., TNF-α mediated increase in DOCK2 expression in HLFs (Qian et al., 2022b). Further studies revealed that the change in the pro-inflammatory phenotype of LF induced by TNF-α was mediated by the PI3K/AKT and NF-κB pathways, which were modulated by DOCK2 (Qian et al., 2022b). In conclusion, inhibition of DOCK2 expression effectively attenuated the inflammatory response, thus alleviating the development of chronic pneumonia. In another model of acute lung injury (ALI),LPS induced DOCK2 expression and reached its highest potent in the most severe lung injury (Xu et al., 2021). Treatment of endotoxin-induced ALI in mice with CPYPP alleviated original inflammatory cell infiltration, thickening of the alveolar wall, lung congestion, and reduced MPO activity, and also effectively reduced the expression and secretion of IL-6, TNF-α, and IL-1β in the lung (Xu et al., 2021). In recent studies, researchers have found that DOCK2 plays an important role in the host immune response to severe acute respiratory syndrome coronavirus 2 (SARS-CoV-2) infection and the development of coronavirus disease 2019(COVID-19) (Namkoong et al., 2022). The COVID-19 infection mainly targets the respiratory system, causing severe pneumonia and damage to lung epithelial cells (Xu et al., 2020). Significant inhibition of DOCK2 expression has been found in COVID-19 patients. In a Syrian hamster model of SARS-CoV-2 infection, inhibition of DOCK2 expression with CPYPP resulted in worsening of pneumonia characterized by weight loss, pulmonary edema, increased viral load, impaired macrophage recruitment, and dysregulation of the type I IFNs response (Namkoong et al., 2022). It is not clear that DOCK2 has a different mechanism between ALI and COVID-19. In general, DOCK2 plays an important role in the respiratory system.

### DOCK2 promotes the occurrence and development of lung cancer

Lung cancer, the deadliest malignancy worldwide, accounts for a large proportion of cancer-related deaths worldwide (Cao et al., 2021; Siegel et al., 20222022). Heterogeneous non-small cell lung cancer (NSCLC) accounts for approximately 85% of all lung cancer cases and includes two major subtypes: Lung squamous cell carcinoma (LUSC) and Lung adenocarcinoma (LUAD) (Molina et al., 2008). Studies show that NSCLC patients with higher lymph node metastasis or cancer stage typically have lower expression of DOCK2. Downregulation of DOCK2 is associated with poorer overall survival in patients with NSCLC (Zeng et al., 2021). Since DNA methylation regulates gene expression or repression in cancer cells, one of the early events and hallmarks in the malignant development of lung cancer is DNA hypermethylation of tumor suppressor genes (Langevin et al., 2015; Li et al., 2016; Pfeifer, 2018). Analysis of NSCLC tissue samples from the cancer genome atlas (TCGA) database versus normal tissue samples revealed that the promoter region of the DOCK2 gene contained higher methylation levels in NSCLC tissue samples and that elevated promoter methylation levels were significantly associated with reduced DOCK2 expression (Zeng et al., 2021). Therefore, the DOCK2 methylation level may serve as a new biomarker for lung cancer detecting. Furthermore, down-regulation of DOCK2 may contribute to the development of non-small cell lung cancer and affect prognostic adverse effects through the activation of the MYC and DNA repair signaling pathways (Zeng et al., 2021). In summary, DOCK2 may play an important role in the development of lung cancer, but the specific mechanism needs to be further explored in depth.

### DOCK2 regulates the inflammatory response of cardiomyocytes and immune rejection of heart transplantation

Myocardial cell injury is associated with the development of many cardiovascular diseases, and apoptosis is an important cause of myocardial injury (Zhang et al., 2018). Analysis by the GSE35182 and GSE53607 databases showed that DOCK2 was significantly upregulated in myocarditis. And increased expression of DOCK2 was positively correlated with pro-inflammatory factors such as IL-6 and TNF-α (Wang et al., 2020). The study revealed a correlation between miR-16 and DOCK2, with miR-16 expression significantly down-regulated in LPS-induced myocarditis. Then miR-16 depletion by inhibitors was followed by an elevated expression of DOCK2 at both mRNA and protein levels (Wang et al., 2020). The miR-16 inhibitor promoted LPS-induced cardiomyocyte apoptosis and attenuated the effect of the miR-16 inhibitor on cardiomyocytes by inhibiting DOCK2 expression, and these phenomena could also be reversed by miR-16mimic treatment (Wang et al., 2020). Furthermore, miR-16 overexpression or miR-16 depletion inhibited or promoted TNF-α, IL-6 and IL-8 expression. These phenomena were reversed by over-expression of DOCK2 or depletion of DOCK2 treatment (Wang et al., 2020). In conclusion, DOCK2 is involved in the development and progression of myocarditis by regulating the expression and secretion of inflammatory factors. On the contrary, miR-16 mimic negatively regulates the expression of DOCK2 to play an antagonistic role. A study in mouse heart transplantation showed a lower inflammatory response in *DOCK2* ^*−/−*^mice, as evidenced by reduced cardiac T-cell infiltration and a reduced number of activated T cells. The *DOCK2* ^*−/−*^mouse recipients that were transplanted with well-preserved myocardial structure showed significantly reduced monocyte infiltration, and few signs of hemorrhage, edema, or necrosis. Thus, DOCK2 deletion effectively attenuated graft rejection, which could synergistically promote permanent graft survival in combination with certain drugs such as Tac (Jiang et al., 2005). DOCK2, a downstream molecule of CD28, led to a decrease in CD25^+^ T cells and the expression of various T cell effector molecules such as IFN-γ, granzyme B, and perforin. DOCK2 deficiency also affected the decreased response of T cells to MHC molecules and the inability to activate Rac properly, thus increasing the survival of allogeneic heart grafts (Jiang et al., 2005). To sum up, DOCK2 promotes allograft rejection through modulation of inflammatory responses and T cell migration.

### DOCK2 mediates the function of macrophages and influences the stability of intestinal flora in the development of colitis

*DOCK2* ^*−/−*^mice have been shown to be more susceptible to colitis caused by *Citrobacter* infection than WT mice, as evidenced by higher mortality, weight loss, *Citrobacter* load, and intestinal damage (Liu et al., 2016). *Citrobacter* infection can induce colitis and requires a combination of innate and acquired immune responses to completely clear the bacteria (Crepin et al., 2016; Mullineaux-Sanders et al., 2019). Compared to WT mice, *DOCK2* ^*−/−*^mice had significant bacterial adherence to mucosal microvilli early in the infection, which partially explains the increased susceptibility of *DOCK2* ^*−/−*^mice to *Citrobacter* infection early in the infection. The CFUs in the liver, mesenteric lymph nodes, and spleen of *DOCK2* ^*−/−*^mice were also significantly higher than those of WT mice in the late stage of infection, suggesting that *Citrobacter* has spread from the mucosal layer into the bloodstream, further leading to systemic dissemination of these bacteria in *DOCK2* ^*−/−*^mice (Liu et al., 2016). Thus, DOCK2 appears to play a role in innate and acquired immunity. However, the exact mechanism of DOCK2 in the defense against colitis needs to be further explored and elaborated. It has been shown that the early inflammatory response in *DOCK2* ^*−/−*^mice can be partially alleviated by reinfusion of WT-BMDM. Thus, DOCK2 plays a role in early colitis by regulating macrophages (Ji et al., 2021). However, the development of colitis is not limited to functional disorders of immune cells, but is also closely related to dysbiosis of the intestinal microbiota. The intestinal flora of both WT and *DOCK2* ^*−/−*^ has been found to be unable to resist *Citrobacter*-induced colitis after removal by antibiotics. After co-caging treatment, WT mice were susceptible to *Citrobacter*-induced colitis. Further analysis of the intestinal flora of WT and *DOCK2* ^*−/−*^mice revealed that *DOCK2* ^*−/−*^mice have a lower group of *Prevotellaceae-NK3B31*, *Lactobacillus* and higher group of *Helicobacter* than WT mice. Therefore, *Prevotellaceae-NK3B31* and *Lactobacillus* may be beneficial for the host in defending against *Citrobacter* infection, while *Helicobacter* can aggravate host susceptibility to *Citrobacter* infection (Xie et al., 2021). So far, DOCK2 alleviates the onset and progression of colitis by regulating the composition of macrophages and microorganisms.

### DOCK2 inhibits colorectal cancer development

Colorectal cancer (CRC) is a highly prevalent cancer, with an incidence rate of approximately 10.0%, the third highest in the world, and a mortality rate of approximately 9.4%, the second highest in the world (Sung et al., 2021). Whole-exome sequencing of 187 genes recurrently mutated or associated with pathway in tumors and blood lymphocytes from 160 CRC patients revealed a correlation between non-silent mutations in the DOCK2 gene and high prevalence (Yu et al., 2015). Meanwhile, recurrent mutations in DOCK2 may led to abnormal activation of RAC1, which promoted overexpression of NF-κB and Wnt/β-catenin pathways, further improving the development of CRC (Myant et al., 2013; Yu et al., 2015). By studying human samples and clinical data from 481 colorectal cancer patients, the researchers found that DOCK2 expression levels were positively correlated with overall survival. Results of clinical data from 160 to 65 CRC patients analysed in public databases (GSE2455120 and GSE2962121) showed similar results. Furthermore, testing 54 CRC patients with positive expression of DOCK2 and 54 control patients revealed that DOCK2 expression levels were positively correlated with CD8^+^ T cell counts and T cells expressing both DOCK2 and CD8 showed the best prognosis in CRC patients. As mentioned above, high expression of DOCK2 was associated with prolonged overall survival and a good prognosis. This result may be due to the fact that high expression of DOCK2 is involved in the recruitment of CD8^+^ T lymphocytes, leading to an increased in the number of CD8^+^ lymphocytes within the tumor center (Miao et al., 2018). TCGA data reveal that DOCK2 is significantly down-regulated in CRC and that this downregulation facilitates tumor escape and spread (Kadkhoda et al., 2021). In general, the expression level of DOCK2 may serve as a new prognostic indicator to help evaluate patients with colorectal cancer and predict different clinical outcomes. Furthermore, targeting DOCK2 may serve as a new therapeutic approach to CRC.

### DOCK2 as a novel marker in prostate cancer

By 2020, prostate cancer (PCa) will be fourth and eighth in global incidence and mortality, respectively (Sung et al., 2021). The lack of effective diagnostic and prognostic biomarkers for PCa makes it difficult to improve prostate treatment rates (James et al., 2016; Kyriakopoulos et al., 2018). Analysis of 4072 samples from the Marmal-aid database, including normal or diseased tissues, revealed that DOCK2 was specifically hypermethylated in PCa tissue samples. To further evaluate its potential as a diagnostic and prognostic biomarker, quantitative methylation specificity analysis PCR of 37 non-malignant and 197 PCa tissue samples from an independent population showed that hypermethylated DOCK2 levels were correlated with PCa (Bjerre et al., 2019). Plasma from 36 healthy subjects, 61 patients with benign prostatic hyperplasia (BPH), 102 patients with limited prostate cancer and 65 patients with early metastatic PCa (MPCA) were examined, and hypermethylated DOCK2 was detected in the plasma of MPCA patients. It was also found that the levels of DOCK2 methylation also increased positively with increasing tumor volume, revealing that hypermethylated DOCK2 may be an independent predictor of MPCA progression (Bjerre et al., 2020). Furthermore, high levels of DOCK2 hypermethylation were significantly associated with disease recurrence rates after radical prostatectomy (Bjerre et al., 2019). DOCK2 plays an important role in the proliferation of PCa cells by mediating the CXCL13/ERK/Akt signaling pathway, which may be a new target for the treatment of PCa (El Haibi et al., 2010; El-Haibi et al., 2012). In summary, DOCK2 has great potential to become a diagnostic and prognostic biomarker for PCa, but its specific mechanisms and signaling pathways in prostate cancer need also to be further explored.

### DOCK2 increases proliferation of chronic lymphocytic leukemia cells

**FIGURE 1:**
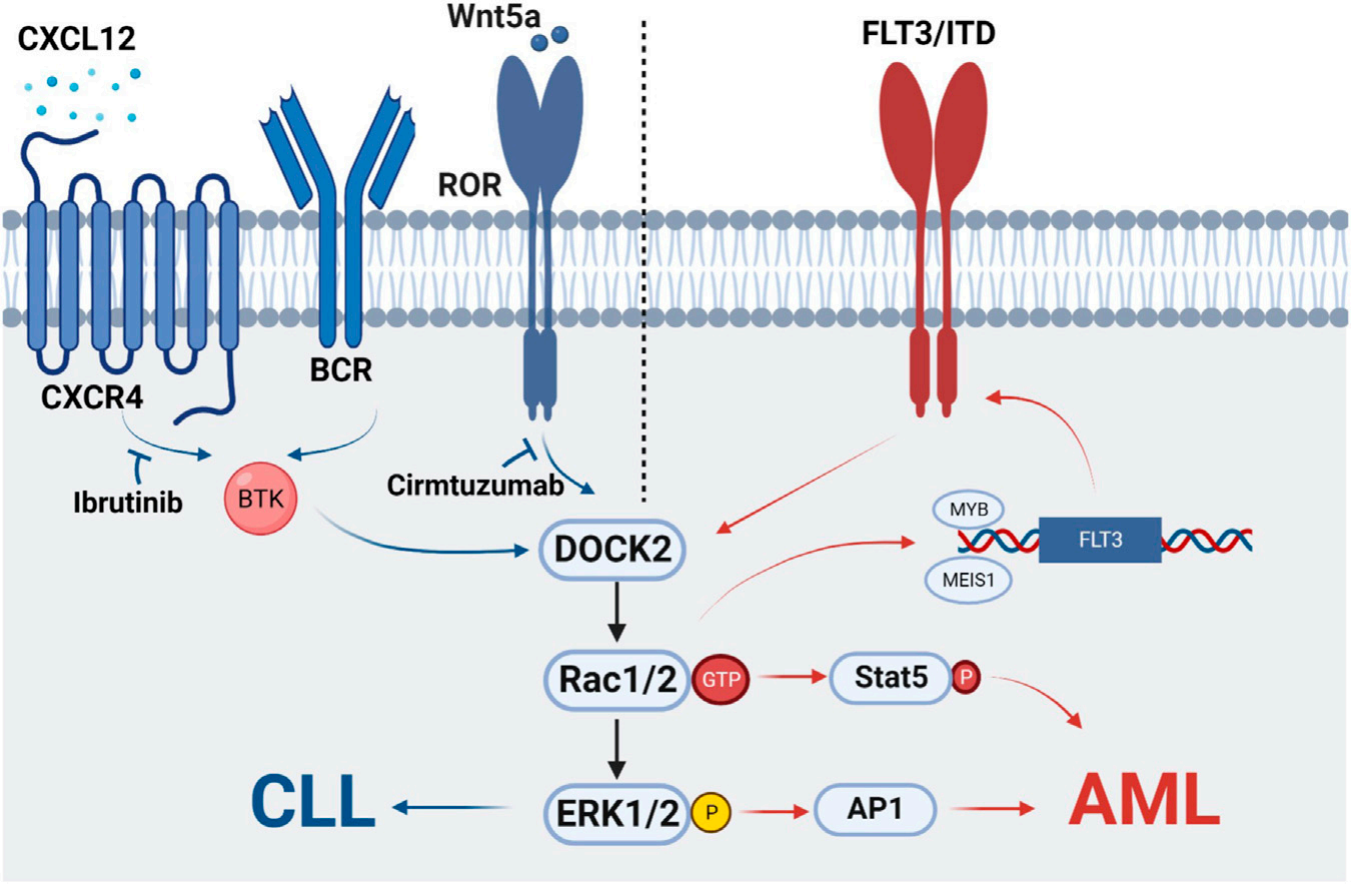
Mechanism of DOCK2 in leukemia.

Chronic lymphocytic leukemia (CLL) is an oncological disease in which lymphocytes accumulate in the bone marrow, lymph nodes, blood, spleen, liver, and other organs. It is characterized by clonal proliferation of immunocompromised, highly differentiated lymphocytes (Zhang and Kipps, 2014; Hallek et al., 2018). In 2010, DOCK2 was found to be significantly expressed in 20 cases of diffuse large B-cell lymphoma and follicular lymphoma (Wang et al., 2010). In addition, a study showed that DOCK2-Rac-ERK pathway is associated with cell proliferation of B-cell lymphoma cell lines Ramos and Raji (Wang et al., 2010; Sakamoto et al., 2017). Tumor formation of DOCK2-KD Ramos cells was also significantly reduced in a xenograft mouse model (Wang et al., 2010; Sakamoto et al., 2017). This is the first time that the important role of DOCK2 in malignancies of the hematopoietic system has been clarified. Current studies have shown that DOCK2-Rac can be activated by two signals that promote the activation of ERK1/2, thus allowing the survival and proliferation of CLL cells. One is that antigens binding to BCR or chemokines (e.g., CXCL12 and CXCR4) binding to Brunton’s tyrosine kinase (BTK) leads to activation of downstream ERK1/2 (O'Hayre et al., 2010; Chen et al., 2016). In addition, the importance of BCR and chemokine receptor signaling is highlighted by the clinical activity of drugs that inhibit BTK, such as ibrutinib (Kipps et al., 2017). The other is that Wnt5a binds to ROR1, allowing ROR1 to interact with DOCK2, thus activating Rac1/2 and EKR1/2 to promote cell proliferation. As both Wnt5a and ROR1 are highly expressed in patients with leukemia, this process can be inhibited by Cirmtuzumab, a monoclonal antibody specific for the extracellular domain of ROR1 (Yu et al., 2016; Hasan et al., 2018). Therefore, the two signaling pathways are inhibited by separate inhibitors without interference, and there are different upstream regulators that together mediate DOCK2 activation and play a role in the development of CLL (Hasan et al., 2021) (Figure 1).

### Undefined role of DOCK2 in acute myeloid leukemia

Acute myeloid leukemia (AML) is an aggressive blood tumor with properties that possess a clonal expansion of myeloid cells. The study reveals FLT3 as the most common mutated gene in acute myeloid leukemia (McKenzie, 2005). More than 30% of patients with AML have FLT3 mutations, and patients with FLT3-ITD mutations, an internal tandem repeat in the proximal membrane region, have a particularly poor prognosis (Levis and Small, 2003; Moreno et al., 2003). When DOCK2 is co-expressed with FLT3 or FLT3-ITD in leukemic cell lines and primary AML samples, DOCK2 interacts with the latter to modulate cell survival. Inhibition of proliferation and colony formation in FLT3-ITD mutant leukemia cells by reducing DOCK2 expression (Wu et al., 2017). Mutations with FLT3-ITD interfered with autophosphorylation and activation of downstream signaling pathways, including PI3K/AKT, RAS/ERK and STAT5 (Wu et al., 2017). Further studies revealed that reduced DOCK2 expression decreased Rac1, ERK and STAT5 activity in leukemia cells bearing FLT3-ITD mutations (Wu et al., 2017). The anti-cancer mechanisms of cytarabine and 5-FU are not the same. Cytarabine (Ara-C) interferes with cell proliferation by inhibiting DNA synthesis, while 5-FU is a pseudo metabolite form that blocks thymidine synthesis. FLT3-ITD leukemia cells showed significantly different therapeutic responses to Ara-C and 5-FU. Notably, MMR-deficient colorectal adenocarcinoma cells were less sensitive to 5-FU and more sensitive to Ara-C. Therefore, the authors assumed that FLT3-ITD and MMR-deficient colorectal adenocarcinoma cells had the same response to both drugs (Wu et al., 2019). In a further study, DOCK2 regulates leukemia cell growth by affecting molecular and protein expression of MMP-related molecules such as MLH1, MSH2, AP1, and DDR factors such as CHK1, WEE1, RAD51, PIM-1, thereby inducing resistance to cytarabine (Wu et al., 2019). Combining treatment with DOCK2 knockdown and DDR inhibitors such as CHK1 inhibitor MK876, WEE1 inhibitor MK1775, and RAD51 inhibitor 1302 significantly enhanced the sensitivity of mice with FLT3-ITD mutant leukemia cells to cytarabine (Wu et al., 2019). This also suggests that DOCK2 may regulate the growth of FLT3-ITD leukemia cells through both MMR and DDR molecular mechanisms (Wu et al., 2019). These results suggest that FLT3-ITD and Rac1 activity synergistically regulate DNA repair activity. The addition of DNA damage response inhibitors to conventional chemotherapy may contribute to the treatment of FLT3-ITD mutations. Thus, targeting DOCK2 may provide a new promising therapeutic target for FLT3-ITD mutations. In addition, stable expression of DOCK2-dCS mutants in Jurkat cells reduced cell attachment and Rac1 activation, demonstrating a link between the dCS of CRK like proto-oncogene, adaptor protein (Crkl) and the SH3 of DOCK2 (Nishihara et al., 2002). Since Crkl enhances the migration of leukemic cells and promotes the development of leukemia (Uemura and Griffin, 1999; Kardinal et al., 2000; Hemmeryckx et al., 2001), the combination of Crkl and DOCK2 may be the one of the therapeutic targets for leukemia (Nishihara et al., 2002). RAS-associated domain family member 2 (RASSF2) is a critical gene for aberrant transcriptional repression in AML (Stoner et al., 2020). RASSF2 was also recently revealed to interact with DOCK2 (Stoner et al., 2020), suggesting that DOCK2 may influence leukemia development *via* multiple pathways. In another study, in 85 patients with AML of unknown etiology, high expression of DOCK2 implied an excellent prognosis for acute myeloid leukemia (Hu et al., 2019). This difference may be related to the different subjects in the two studies, as the 85 patients were not specifically explored for the presence of the FLT3-ITD mutation (Hu et al., 2019). Therefore, it is not well known whether FLT3 is highly expressed or FLT3-ITD mutations are highly expressed in patients receiving chemotherapy, nor is genome-wide information on these 85 patients. In summary, it is still necessary to delve further into the exact role that DOCK2 plays in all types of AML.

### DOCK2 promotes melanoma stem cell activity

Melanoma is a malignant tumor caused by abnormal proliferation of melanocytes. It is more likely than other types of skin cancer to invade nearby tissues and spread to other parts of the body. The key molecular and underlying mechanisms of the development and metastasis of melanoma are still poorly understood (Lemos et al., 2018; Xu et al., 2019). A recent study has revealed that DOCK2 plays an important role in the malignant development of tumors by regulating melanoma stem cell activity. In melanoma stem cells, ADAR2 serves as a gene encoding an editing enzyme required to maintain melanoma stem cells. ADAR2 increases the stability of DOCK2 mRNA and promotes the activation of Rac1, which phosphorylates Akt and NF-κB. Further upregulation of the stemness-associated genes SOX2, ALDH1, OCT3/4, C-MYC and the anti-apoptotic gene BCL-2 expression, leading to tumor formation in melanoma stem cells (Zhang et al., 2022). Another study also confirmed that DOCK2 is regulated by the hnRNP A2B1 variable shedder, thus promoting the malignant development of melanoma by helping melanoma stem cells evade the normal apoptotic process *in vivo* and *in vitro* (Chu et al., 2021). hnRNP A2B1 is an important splicing factor that binds to specific RNAs and regulates their post-transcriptional processing, thereby affecting RNA expression and tumor progression (Chu et al., 2021). Overall, DOCK2 promotes the proliferation of melanoma stem cells and inhibits stem cell apoptosis, thus promoting the development, metastasis, and recurrence of melanoma. Therefore, DOCK2 could be a candidate target for the clinical treatment of melanoma.

### The role of DOCK2 in human combined immunodeficiency disease

Combined immunodeficiencies (CIDs) are manifested as a combination of antibody immunodeficiency and cellular immunodeficiency, characterized by impaired quality or function of T cells and impaired antibody-mediated responses (Al-Herz et al., 2014). Five infants with double allele mutations in DOCK2, which are accompanied by aggressive bacterial and viral infections, were identified in 2015. The main manifestation was a significant reduction in the number and function of immune cells. These patients had lower numbers of T and B cells in the blood and defective IgG antibody production compared to healthy infants. In addition, they have a defect in NK cell degranulation, resulting in their decreased cytotoxicity (Dobbs et al., 2015). These abnormal manifestations were also very similar in *DOCK2* ^*−/−*^ mouse-related experiments (Kunimura et al., 2020). Clinical and genetic characteristics of a DOCK2-deficient patient were reported in 2017 (Alizadeh et al., 2018). DOCK2 deficiency is a congenital immunodeficiency and a rare autosomal recessive combined immunodeficiency presenting with very early onset, severe bacterial and viral infections (Sharifinejad et al., 2021). The patient had T cell lymphopenia and reduced numbers of B cells and NK cells. Meanwhile, the patient had elevated levels of IgM expression and cytomegalovirus infection (CMV). In addition, the DOCK2-deficient girl had low levels of T cell receptor excision circle (TREC) expression. The presence of functionally normal T cells in a rare patient with CID has been reported and the girl was considered to suffer from CID. DOCK2-deficient patients were reported in 2019 and three patients with DOCK2 deficiency were reported in 2021 (Alosaimi et al., 2019; Sharifinejad et al., 2021; Aytekin et al., 2021; D'Astous-Gauthier et al., 2021). DCOK2 deficiency patients presented with severe compound immune deficiency and the CD4^+^ T cell lymphopenia manifest in all DOCK2-deficient patients studied to date. In conclusion, DOCK2 plays an important role in immunodeficiency disease and genetic testing is necessary for early diagnosis of DOCK2 deficiency or mutation.

## Discussion

**FIGURE 2:**
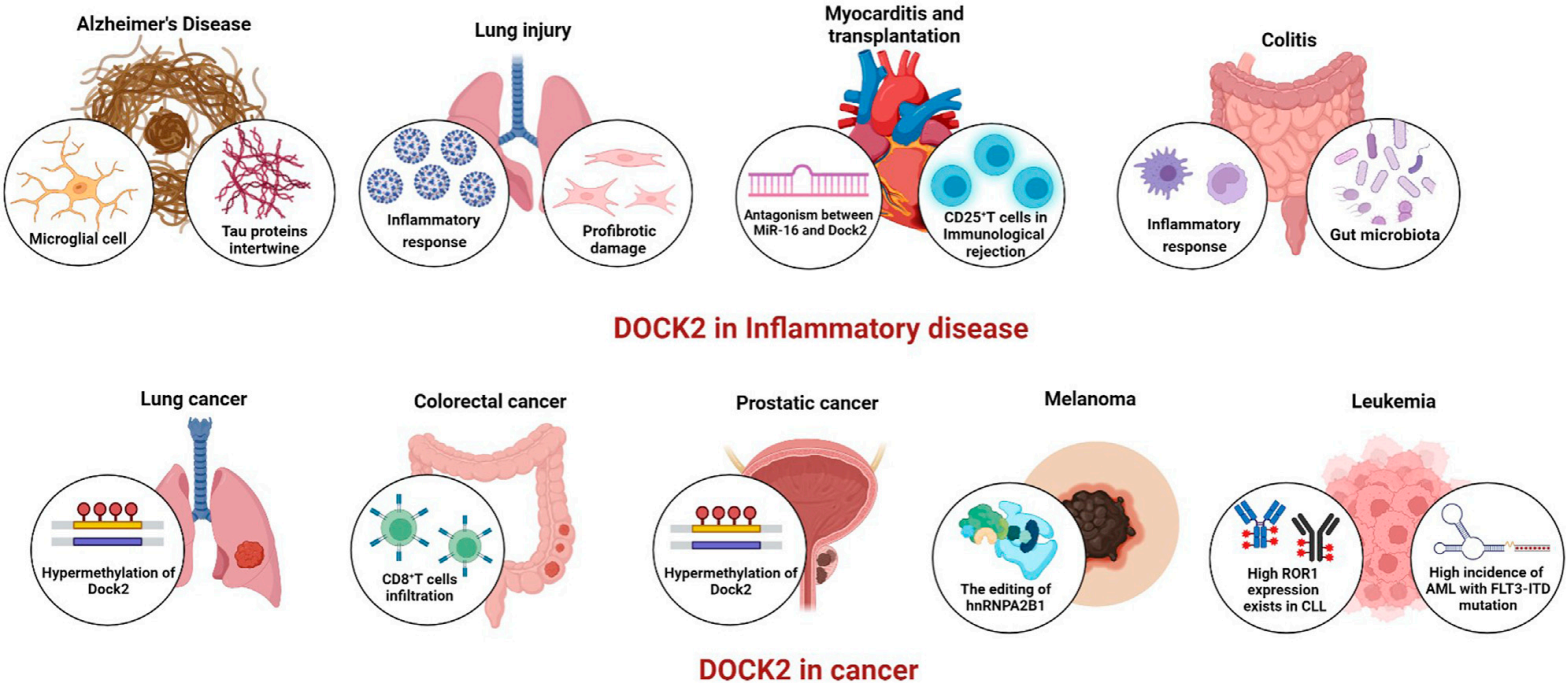
The role of DOCK2 in diseases.

DOCK2 belongs to the DOCK-A subfamily together with DOCK1 and DOCK5, which can activate the small G protein Rac but not Cdc42. Current studies show that DOCK2 mainly regulates Rac, but it affects different functions in innate and adaptive immune cells. Overall, although the functional regulation of DOCK2 by various immune cells varies and the specific mechanisms involved in the innate and adaptive immune processes remain unclear, it significantly affects the normal function of immune cells. Until now, 13 cases of the autosomal recessive mutation of DOCK2 have been reported. The earliest reported were for combined immunodeficiency with early-onset invasive viral and bacterial infections. DOCK2 deficiency is a disease with a very poor prognosis in patients who do not undergo hematopoietic stem cell transplantation (HSCT) (D'Astous-Gauthier et al., 2021). Cells from DOCK2-deficient patients exhibit multiple defects, including lymphocyte chemotactic responses, degranulation of NK cells, ROS production by neutrophils, and type I interferon production by peripheral blood monocytes (Kunimura et al., 2020). DOCK2 deficiency in a patient with hyper IgM phenotype or early-onset invasive infections both show that DOCK2 plays a crucial role in human immune cells (Dobbs et al., 2015; Alizadeh et al., 2018). DOCK2 expression contributes to the formation of Aβ plaques in the cerebral cortex and hippocampus of AD transgenic mouse models (Cimino et al., 2009; Cimino et al., 2013). Reducing Aβ plaque formation *in vivo* and inhibiting microglia-mediated nonspecific immune responses may be more beneficial than enhancing microglial phagocytosis. In lymphocytes, downregulation of DOCK2 expression using sh-RNA leads to decreased proliferation of B-cell lymphoma cells, a mechanism related to DOCK2-mediated Rac-ERK (Wang et al., 2010; Sakamoto et al., 2017). The expression of DOCK2 is also not restricted to immune cells. As mentioned above, researchers have reported that DOCK2 plays an important role in fibroblasts and influences in the development and progression of pulmonary fibrosis. In addition, DOCK2 is involved in the antiviral immune response in SARS-CoV-2 infection and influences the process of SARS-CoV-2. COVID-19 caused by SARS-CoV-2 is a serious global public health issue. DOCK2 plays an important role in the host immune response to SARS-CoV-2 infection and may be further explored as a potential biomarker and/or therapeutic target (Namkoong et al., 2022). Inhibition of DOCK2 expression by a specific siRNA in MCE1-ROR1 cells inhibits Wnt5a-induced ERK1/2 phosphorylation (Hasan et al., 2021). However, it is unclear whether DOCK2 must be phosphorylated to activate Rac1/2 and subsequently EKR1/2. It provides a theoretical basis for the clinical evaluation of antibodies alone or in combination with other inhibitors or other targeted therapies for patients with CLL or other ROR1-expressing malignancies. In AML, FLT3-ITD interacts with DOCK2 to activate Rac1/2 and regulate DNA repair activity. The addition of DNA damage response inhibitors to conventional chemotherapy may contribute to the treatment of FLT3-ITDAML, revealing DOCK2 as a promising therapeutic target and providing a new strategy for the treatment of aggressive tumors (Wu et al., 2019). To date, DOCK2 has been closely related to neoplastic diseases (Figure 2). DOCK2 has been identified to be associated with prognostic factors in a variety of cancer types, such as acute myeloid leukemia, prostate cancer, colorectal cancer, and non-small cell lung cancer, indicating that DOCK2 is a promising therapeutic target. It is possible to detect the expression level of DOCK2 in patients with different oncological disorders, such as the high methylation of DOCK2 in lung cancer and prostate cancer, to further understand the mechanisms associated with the diseases and to provide new therapeutic targets for the treatment of the diseases. It is also possible to monitor the effectiveness of treatment, providing a new direction for attacking these diseases in the future. A comprehensive understanding of DOCK2 may help determine which patients are more likely to benefit from clinical treatment. Therefore, it is essential to further explore the therapeutic potential of DOCK2 as a critical molecule involved in the inflammatory process and as a suitable candidate for therapeutic manipulation. Future studies can shed lighter on the molecular pathways associated with DOCK2 in various disease states to promptly select appropriate targets for therapeutic interventions, bringing more expectations and new hopes for disease placement.

## Glossary

AD: Alzheimer’s disease
AML: acute myeloid leukemia
APC: antigen-presenting cells
BMDM: bone marrow-derived macrophages
CCR7: chemokine receptor 7
COX: cyclooxygenase
CXCL13: C-X-C motif chemokine 13
CPYPP: 4-[3'-(2″-chlorophenyl)-2′-propen-1′-ylidene]-1-phenyl-3,5-pyrazolidinedione
CRC: colorectal cancer
CLL: chronic lymphocytic leukemia
Crkl: CRK like proto-oncogene, adaptor protein
CIDs: Combined immunodeficiencies
CMV: Cytomegalovirus infection
DOCK2: Dedicator of cytokinesis 2
DHR1: DOCK homology region-1
ELMO1: engulfment and cell motility1
ER: endoplasmic reticulum
FMT: fibroblast to myofibroblast
GEFs: guanine nucleotide exchange factors
HFHF: high-fat and high-fructose
HSCT: hematopoietic stem cell transplantation
Ig: immunoglobulin
IPF: idiopathic pulmonary fibrosis
IFN: types of interferon
LFs: lung fibroblasts
LPS: lipopolysaccharide
MesoMT: mesothelial to mesenchymal transition
MZ: marginal zone
NT: Naïve T
NK: Natural killer
ROS: reactive oxygen species
PTEN: phosphatase and tensin homolog deleted on chromosome 10
pWASP: Phosphorylated Wiskott–Aldrich syndrome protein
PBR: polybasic amino acid region
PIP3: phosphatidylinositol 3,4,5-trisphosphate
PI3K: phosphatidylinositol 3-kinase
PH: Pleckstrin homology
SH: Src Homology
S1P: sphingosine-1-phosphate
WT: wild-type
Tg: transgenic
TCR: T cell receptors
LEF-1: lymphoid enhancer-binding factor 1
HIF-1α: hypoxia-inducible factor-1α
NKG2D: natural killer group 2 member D
MHC: major histocompatibility complex
ERK: extracellular signal-regulated kinase
GPCR: G protein-coupled receptor
NETs: Neutrophil extracellular traps
fMLP: N-formylmethionyl-leucyl-phenylalanine
IKKβ: IκB kinaseβ
MPO: myeloperoxidase
HFD: high-fat diet
CLRs: C-type lectin receptors
mDCs: myeloid dendritic cells
pDCs: plasmacytoid dendritic cells
TLRs: Toll-like receptors
IRF-7: Interferon Regulatory Factor 7
PGE2: prostaglandin E2
TGF-β: transforming growth factor-β
HLFs: human lung fibroblasts
ALI: cute lung injury
COVID-19: coronavirus disease 2019
SARS-CoV-2: severe acute respiratory syndrome coronavirus 2
LUSC: lung squamous cell carcinoma
NSCLC: non-small cell lung cancer
LUAD: lung adenocarcinoma
TCGA: The Cancer Genome Atlas
PCa: prostate cancer
BPHbenign prostatic hyperplasiaMPCa: benign prostatic hyperplasiaMPCametastatic PCa
TREC: T cell receptor excision circle (TREC)
